# A Dynamic Threshold Cancellation Technique for a High-Power Conversion Efficiency CMOS Rectifier

**DOI:** 10.3390/s21206883

**Published:** 2021-10-17

**Authors:** António Godinho, Zhaochu Yang, Tao Dong, Luís Gonçalves, Paulo Mendes, Yumei Wen, Ping Li, Zhuangde Jiang

**Affiliations:** 1 Chongqing Key Laboratory of Micro-Nano Systems and Smart Transduction, Collaborative Innovation Center on Micro-Nano Transduction and Intelligent Eco-Internet of Things, Chongqing Key Laboratory of Colleges and Universities on Micro-Nano Systems Technology and Smart Transducing, National Research Base of Intelligent Manufacturing Service, Chongqing Technology and Business University, Chongqing 400067, China; godinhoa2208@gmail.com (A.G.); zhaochu.yang@ctbu.edu.cn (Z.Y.); zdjiang@mail.xjtu.edu.cn (Z.J.); 2 Department of Microsystems (IMS), Faculty of Technology Natural Sciences and Maritime Sciences, University of South-Eastern Norway, 3616 Kongsberg, Norway; 3 Center for MicroElectromechanical Systems (CMEMS-UMinho), University of Minho, 4800-058 Guimarães, Portugal; lgoncalves@dei.uminho.pt (L.G.); paulo.mendes@dei.uminho.pt (P.M.); 4 School of Electronic Information and Electrical Engineering, Shanghai Jiao Tong University, Shanghai 200240, China; yumei.wen@sjtu.edu.cn (Y.W.); liping_sh@sjtu.edu.cn (P.L.)

**Keywords:** vibration energy harvester, power management circuit, CMOS rectifier, dynamic threshold cancellation technique, high power conversion efficiency

## Abstract

Power conversion efficiency (PCE) has been one of the key concerns for power management circuits (PMC) due to the low output power of the vibrational energy harvesters. This work reports a dynamic threshold cancellation technique for a high-power conversion efficiency CMOS rectifier. The proposed rectifier consists of two stages, one passive stage with a negative voltage converter, and another stage with an active diode controlled by a threshold cancellation circuit. The former stage conducts the signal full-wave rectification with a voltage drop of 1 mV, whereas the latter reduces the reverse leakage current, consequently enhancing the output power delivered to the ohmic load. As a result, the rectifier can achieve a voltage and power conversion efficiency of over 99% and 90%, respectively, for an input voltage of 0.45 V and for low ohmic loads. The proposed circuit is designed in a standard 130 nm CMOS process and works for an operating frequency range from 800 Hz to 51.2 kHz, which is promising for practical applications.

## 1. Introduction

Presently, energy harvesting appears as a promising reliable technology that can prolong the lifetime of batteries and power wireless sensor networks (WSNs) for environmental monitoring [1]. However, in these WSN applications, ambient vibrations are unpredictable, time-varying, and low amplitude, which restricts the available power of the energy harvesting system [2]. To overcome these drawbacks, research groups have been focusing on using piezoelectric harvesters due to their high power density and capability to integrate MEMS and CMOS technology, making it possible to develop all the systems (energy harvester and electronic system) in a single chip [3,4,5]. Thus, to maximize the amount of energy transferred under different ambient conditions, a power management circuit (PMC) is crucial in order to extract, convert, store, regulate, and manage the scavenged energy from the piezoelectric device [6,7].

Because the vibrational energy sources produce AC signals, scavenging such energy requires a full-wave rectifier as a key circuit inside the PMC, which allows the AC/DC conversion to properly power the WSNs. However, because the output power of the vibrational energy harvester is low [4], the high forward voltage required by standard full-wave diode bridges and Schottky diode rectifiers limits their use on these low power restrict applications [8]. To surpass these limitations, diode-connected MOS transistors have been widely used because they present similar I-V characteristics to the standard diodes. Thus, designing the rectifier in CMOS technology is highly desirable to decrease the device’s form factor and easily integrate with the energy harvester while exploring new dynamic techniques to reduce the power consumption, achieve high PCE, and minimize leakage current [9,10].

Recent work has been developing dynamic threshold techniques to reduce the threshold voltage effect [11]. Addressing these techniques allows for the reduction of total voltage drop and mitigation of the reverse leakage current in the active stage. By attending to these concerns during the design of the circuit, it is possible to minimize the circuit’s overall power and leakage current consumption. Thus, all these conditions were carefully considered during the design of the proposed high-power efficiency CMOS rectifier to attend to the demands of this application.

In this work, a new CMOS rectifier structure for piezoelectric energy harvesters is presented. It combines a passive stage negative voltage converter (NVC) with an active diode controlled by a dynamic threshold cancellation circuit to build a new architecture that can reduce its total voltage drop. With this configuration, a voltage drop lower than 2 mV can be achieved in the second stage, which consequently enhances features such as VCE and PCE, as well as reduces the reverse leakage current that flows from the load.

## 2. CMOS Rectifiers

### 2.1. Passive Rectifiers

The CMOS gate cross coupled can replace the conventional full-wave bridge rectifier to overcome the high forward voltage drop because it allows a minimum input voltage to operate [10,12,13]. However, this topology still lacks efficiency due to the threshold voltage ($V_{TH}$) drop across the diode connected in each conduction path [13].

The fully cross-coupled rectifier intends to fulfill the gap of the previous configuration by eliminating all $V_{TH}$ drops, which reduces the voltage drop across this stage [13]. Consequently, this topology improves both PCE and VCE of the circuit [13]. However, the reverse leakage current appears to be the main disadvantage of using this single configuration, which affects the power transferred from the circuit to the load [10]. Thus, an extra circuit must be added to overcome this issue.

### 2.2. Active Rectifiers

To prevent the circuit from reverse leakage current, the CMOS passive rectifiers combined with an active configuration can mitigate the reverse leakage current to enhance the DC power of the load [14,15,16,17,18,19]. In these active configurations, comparators are designed to control the gate voltage of the active diode (or so-called the main transistor) depending on its input and output voltage conditions. In work done by Peters et al. [15], an active rectifier with a bulk-input comparator technique is proposed for ultra-low-voltage energy harvesting systems. However, when the input voltage is higher than the output voltage, the PN junctions between the bulk and source terminal of the input transistor will be turned on. Consequently, the reverse leakage current will flow from the cathode terminal to the anode terminal through the body PN junctions, which compromises the efficiency of the circuit [8]. In addition, the proposed rectifier in [19] has a frequency range not suitable for the application of this research work. In contrast, in the following research papers [8,9,20,21], the frequency bandwidth corresponds to the desired application. The authors use two active diodes to control the reverse current that flows through the two NMOS in each input cycle, and two PMOS in cross-coupled to provide the conduction path. However, the dynamic range does not meet the requirements to achieve a high PCE for input voltages lower than 1 V, which is critical for energy harvesting applications [8]. In [20], the authors designed a fully active configuration using PMOS and NMOS to ensure that the reverse current through the PMOS input source is zero. The main disadvantage of this configuration occurs when the two NMOS devices turn on simultaneously, which leads to power losses. Chang et al. [21] proposed a rectifier with a third comparator to eliminate the oscillations of NMOS, which avoids the two active diodes turning on/off simultaneously. Nevertheless, the PCE is only high for an input voltage around 4.88 V.

However, the main limitations of these configurations are that they cannot control the $V_{G}$ of the main transistor to increase $V_{SG}$ during the conduction phase. Thus, it is not possible to reduce the internal resistance of this transistor, which limits the output power of the rectifier. Therefore, an extra circuit is needed to reduce the threshold voltage effect of this transistor to overcome these drawbacks.

### 2.3. Threshold Cancellation Topologies

Several threshold cancellation topologies were proposed to enhance the output stored voltage by dynamically reducing the threshold voltage effect of the main transistor of the rectifier [22,23,24,25]. The threshold voltage is a process parameter dependent on the oxide type and thickness [24]. Low threshold voltage MOSFETs present a high leakage current caused by the low substrate doping, which leads to an increase in power consumption and reliability problems [24,26]. Thus, these threshold cancellation techniques are used to avoid those types of MOSFETs since it is only needed to reduce the threshold voltage effect when the main pass transistor is ON. In [25], a low-voltage CMOS rectifier is proposed to perform this technique by using the bootstrap technique, which has enhanced the output voltage stored in the load capacitor. However, for the minimum operating voltage of this configuration (0.8 V), the PCE of this circuit is around 30%, which is not enough for the requirements of this application.

An active bootstrapping rectifier is presented in [27] to overcome the issues of the previous work. This topology uses two active diodes to control the conduction path for each input cycle and a bootstrap technique to reduce the threshold voltage of both main pass PMOS. Additionally, an adaptive voltage converter is set in this work to adjust the gate voltage of the main pass PMOS, which reduces the voltage drop by reducing the on-resistance. Besides lowering the reverse leakage current, the PCE of this configuration can still be improved for input voltages smaller than 1 V. To overcome the low PCE values for a narrow input voltage range, in [28], a dual switching technique replaced the two active diodes. This approach can maintain a constant gate bias on the two main NMOS transistors, avoid the reverse leakage current, reduce the area on-chip, and enhance the PCE for low voltage applications. However, high values for PCE can only be obtained for input frequencies around 20 kHz, which makes the frequency bandwidth narrow.

## 3. Design Implementation

Regarding the inherent output characteristics of the piezoelectric transducer, the proposed CMOS rectifier was mainly designed to achieve a high PCE for wide low input voltage and frequency conditions. Therefore, the operational voltage ranges from 0.4 V to 1 V, and the working frequency varies from hundreds of Hz to a few kHz. In addition, the output impedance of the energy harvester is not considered in this design because the matching impedance process is performed before this rectification stage in the PMC. Thus, the main goal of this work is to reduce the voltage drop across the structure by applying a threshold cancellation technique that will further enhance the power converted to the ohmic load. These improvements will overcome the drawbacks of previous work by mitigating the reverse leakage current, and thus enhancing the PCE for a low input voltage range.

**Figure 1:**
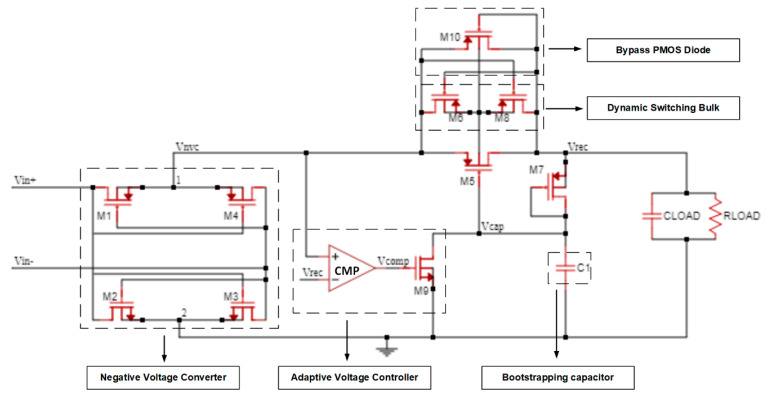
Schematic of the proposed active rectifier composed by a NVC and an active diode controlled by a threshold cancellation circuit.

Figure 1 shows the simplified schematic of the proposed active rectifier. It consists of an NVC and an active diode biased by a threshold cancellation circuit. The first stage is set to perform the signal full-wave rectification. However, because this passive stage cannot control the reverse current from the load capacitor when the output voltage is higher than the input, a second stage active diode (M5) is needed. This active stage is composed of a PMOS controlled by a threshold cancellation circuit with a bootstrapping capacitor to reduce the effective threshold of the active diode, and an adaptive voltage controller (AVC) to adjust the gate voltage of M5 by controlling the charging/discharging cycle of the bootstrapping capacitor. To perform it, a two-input common gate comparator and an NMOS transistor are used. Besides these stages, a dynamic switching bulk (DSB) technique was used to control the bulk voltage of the active diode PMOS.

### 3.1. Negative Voltage Converter

The first stage is fully passive, and it is used to perform the signal full-wave rectification by applying a fully-cross coupled configuration. During the positive half period of the input signal ($V_{in+}>V_{in-}$), M1 and M3 will be conductive as soon as the input voltage gets larger than $V_{THn}$ and $\left|V_{THp}\right|$. In this cycle, node 1 is connected to $V_{in+}$ and node 2 to $V_{in-}$. For the negative period of the sine wave, M2 and M4 are conducting while the previous two transistors are now turned off (cut-off region). Therefore, the higher voltage potential is always at $V_{nvc}$, whereas the lowest potential is at 0 V. The voltage drop of the NVC is given by $V_{DSn}+V_{SDp}$ in each conduction path, where $V_{DSn}$ and $V_{SDp}$ are the voltage drop of NMOS transistors M2 or M3 and PMOS transistors M1 or M4, respectively.

To meet all the power restrictions related to the piezoelectric energy harvesting systems, the rectifier circuit must minimize the voltage drop across the rectification process. As less voltage drop occurs, both the VCE and the PCE of the circuit will be higher. For this stage, NVC, the main requirement is to decrease the voltage drop associated with each MOSFET by reducing their on-resistance.

### 3.2. Active Diode

One of the main challenges on the rectifier circuit is to avoid the reverse leakage current by controlling the operation of transistor M5. Therefore, an active diode controlled with a threshold cancellation circuit can regulate the work behavior of this device depending on the voltage potential between the input and output. The deployed threshold cancellation circuit controls the gate potential of the MOSFET M5 by comparing the input/output voltage conditions. Additionally, the width of M5 has a large influence on the performance of this rectifier because the voltage drop is mainly affected by this parameter due to the internal on-resistance. Consequently, since the gate capacitance of M5 depends on the width, the turn on/off time of the transistor will also be affected by this parameter. In addition, the DSB technique, composed of M6 and M8, is deployed to reduce the leakage current through the bulk terminal of M5 by connecting it to the higher potential ($V_{nvc}$ or $V_{rec}$). Another advantage of this technique is eliminating the body effect of M5, which reduces the rectifier voltage drop. Both M6 and M8 can be small in size since only a very low current flows through them during the start-up phase.

To assure a safe start-up of M5, a bypass PMOS diode (M10) was connected in parallel. This transistor makes the active diode more robust by preventing it from leakage current in the subtraction that induces latch-up. After the start-up phase, the bypass diode always operates in the cut-off region.

### 3.3. Threshold Cancellation Circuit

In order to reduce the threshold voltage effect on M5, a bootstrap technique is used by attaching the capacitor $C_{1}$ to the output terminal. When the $V_{NVC}$ is higher than the output voltage $V_{rec}$, M5 is turned ON, since $V_{SG5}$ is no longer lower than $V_{TH5}$, and thus it can be defined in (1). Nevertheless, because M5 is operating in the deep-triode region due to $V_{SD5}\ll 2\cdot \left(V_{SG5}-\left|V_{TH5}\right|\right)$, $V_{SG5}$ can also be defined according to the on-resistance equation, see (2).

(1) $$V_{SG5}=V_{NVC}-V_{CAP}\;$$

(2) $$V_{SG5}=\frac{1}{{µ}_{p}\cdot C_{ox}\cdot W_{5}/L_{5}\cdot R_{SD5}}+\left|V_{TH5}\right|$$

Here, ${µ}_{p}$ is the carrier mobility, $C_{ox}$ is the oxide capacitance, $W_{5}/L_{5}$ is the aspect ratio of transistor M5, and $V_{TH5}$ is its respective threshold voltage.

The bootstrapping capacitor ($C_{1}$) is charged up through an auxiliary diode-connected PMOS transistor M7, and it maintains a value when the rectifier is under the steady-state regime. At this time, because $C_{1}$ is discharging, $V_{CAP}$ is one diode forward-bias voltage ($V_{TH7}$) bellow $V_{rec}$ due to M7 is being in the saturation region. Thus, the voltage held on the bootstrapping capacitor can be defined as:

(3) $$V_{CAP}=V_{rec}-\left|V_{TH7}\right|$$

$V_{SG5}$ and $V_{CAP}$ from (2) and (3), respectively, can be replaced in (1), which means that $V_{rec}$ can now be defined according to the following equation:

(4) $$V_{rec}=V_{NVC}-\left(\left|V_{TH5}\right|-\left|V_{TH7}\right|\right)-\frac{1}{{µ}_{p}\cdot C_{ox}\cdot W_{5}/L_{5}\cdot R_{SD5}}$$

According to (4), the rectified signal is highly influenced by the size of M5 and the threshold voltage of both M5 and M7, and thus it is vital to manage these parameters to enhance the output signal voltage. The implemented threshold cancellation circuit reduces the voltage drop of the main pass transistor M5 by lowering the threshold voltage effect. Additionally, the size of the bootstrap capacitor is an important design concern for the implementation of the proposed rectifier. Integrated capacitors consume a large area on the chip when standard CMOS processes are used [24]. Therefore, $C_{1}$ was set at 200 fF not only to reduce the correspondent area on the die but also to have a faster charging/discharging time. Consequently, this low bootstrap capacitance allows a lower gate voltage of M5 at the ON state. Due to the reduction of its internal source to drain resistance, the voltage drop is decreased. The reverse leakage current during the OFF state will be avoided because $V_{SG5}$ is reduced. Moreover, it is necessary to have an auxiliary circuit to hold the $V_{CAP}$ node when M5 is OFF, and to discharge it at the opposite state.

The bootstrapping capacitor is used to reduce the threshold voltage effect of M5. However, an increase in its on-resistance can be noticed due to the reduction of $V_{SG5}$. Thus, a conduction path needs to be generated to discharge the gate of M5 during the ON state, which will lead to a further increase of $V_{SG5}$. The proposed AVC is composed of NMOS M9 and a comparator CMP that drives its gate. When $V_{NVC}$ is higher than the output voltage $V_{rec}$, the comparator CMP should immediately turn on M9 to provide a discharge path of the $V_{CAP}$ node. Consequently, it will turn on the main pass transistor M5 with a low on-resistance. Because the large size of M5 increases the gate capacitance, the AVC must have a faster bias signal control to switch the discharge path of the gate node ($V_{CAP}$). Thus, the comparator must be designed to attend to these demands.

**Figure 2:**
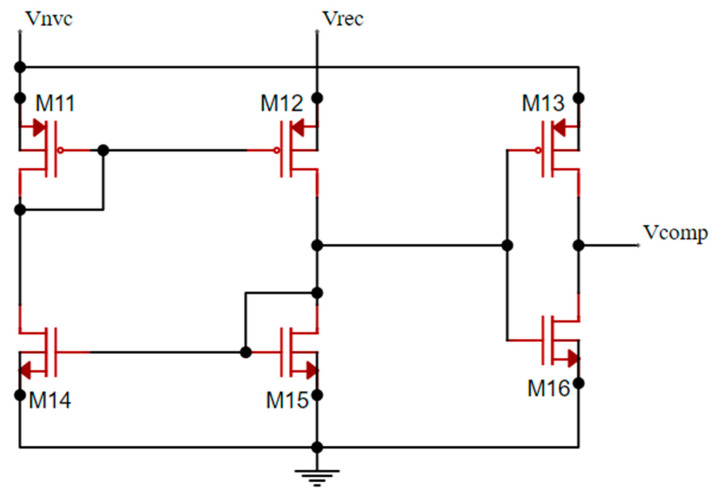
Schematic of the two-input common gate comparator CMP.

Figure 2 shows the proposed two-input common gate comparator. This comparator is composed of a current mirror stage to make the comparison, plus an inverter block to bias the gate of M9. Even if the transistor of the current mirror should be as small as possible to reduce the current consumption of the comparator, the size of M12 and M15 must be carefully chosen to manage the delay, and consequently, the reverse leakage current in M5. These two transistors cannot have the same W/L ratio as M11 and M14. Otherwise, this would generate a delay caused by the inverter’s gate capacitance’s low charging/discharging time. Additionally, they cannot be much larger than the other transistors because of the reduced time that M5 would be ON, which would lead to a PCE reduction. Therefore, M12 and M15 only need to be slightly higher to provide the required charging/discharging time to reduce the delay of the overall comparator. Table 1 summarizes the dimension values of the proposed rectifier circuit.

## 4. Results and Discussion

**Figure 3:**
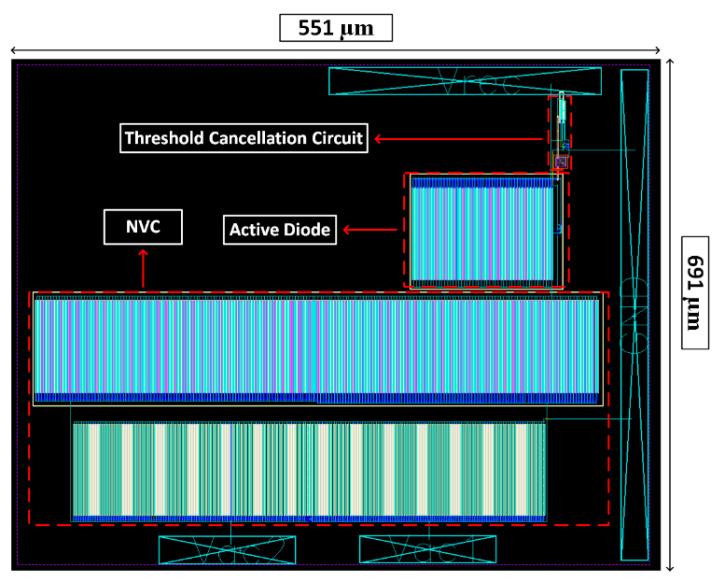
Physical layout of the proposed CMOS rectifier.

The simulation experiments were carried out using Cadence Virtuoso Analog Design Environment with a 130 nm CMOS process. The respective physical layout of the CMOS rectifier is presented in Figure 3. To replicate the output behavior of the energy harvester, the default input sinusoidal voltage amplitude and frequency used in the simulations were 600 mV and 3.2 kHz, respectively. Throughout most of the tests, $C_{LOAD}$ and $R_{LOAD}$ were set at 2 µF and 5.5 kΩ to simulate the capacitance of the storing capacitor and the impedance of the electronics to be powered, respectively.

### 4.1. Transient Behavior

**Figure 4:**
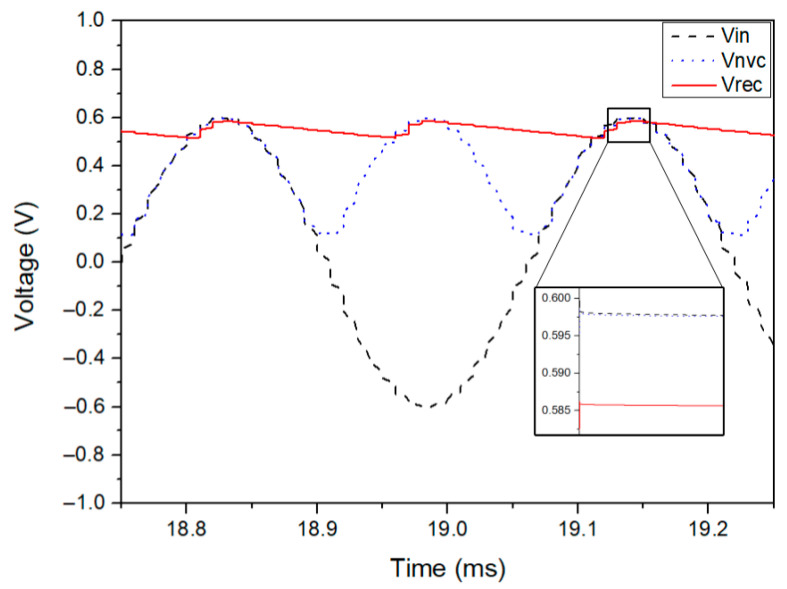
Simulated waveforms of the rectifier for $R_{\mathrm{LOAD}}=5.5\;kΩ$ and $C_{\mathrm{LOAD}}=2\;µF$.

The transient performance of the output voltage, in both stages, is displayed in Figure 4. The first stage performs the full-wave rectification by converting the negative input voltages ($V_{IN}$) into positive ones ($V_{NVC}$). The voltage drop on this stage is around 1 mV, whereas the total voltage drop on the circuit is around 12 mV, which is possible due to the reduction of the internal resistance of the main pass transistor M5. The achieved voltage drop is crucial to enhance the output voltage across the load.

**Figure 5:**
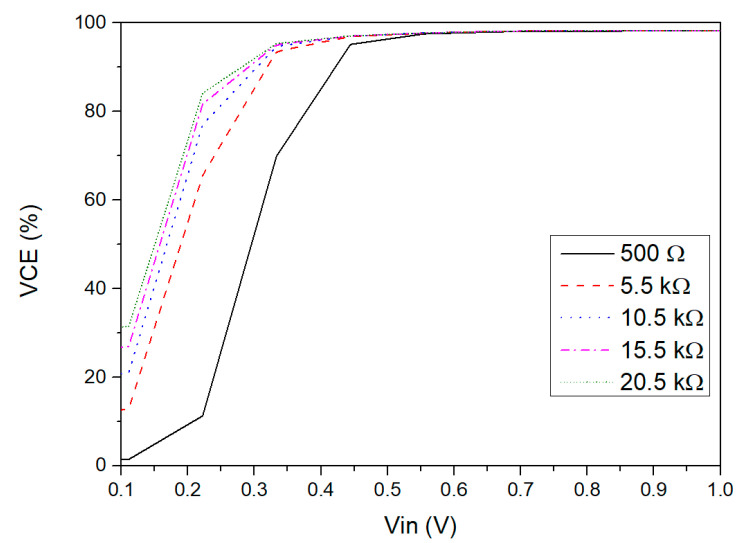
VCE versus input voltage amplitude simulated for different ohmic loads.

Figure 5 shows the VCE behavior versus the input voltage amplitude for different $R_{LOAD}$ values. It is possible to observe that the proposed rectifier can work efficiently for an input voltage range from 0.45 V to 1 V for different ohmic loads, with a VCE varying between 96% and 99%. For an input voltage lower than 0.4 V, the VCE sharply decreases because the NVC transistors will enter the subthreshold region or even cut-off. Moreover, it can be noticed that the rectifier VCE is higher for larger load resistors, as would be expected.

### 4.2. Reverse Leakage Current Analysis

**Figure 6:**
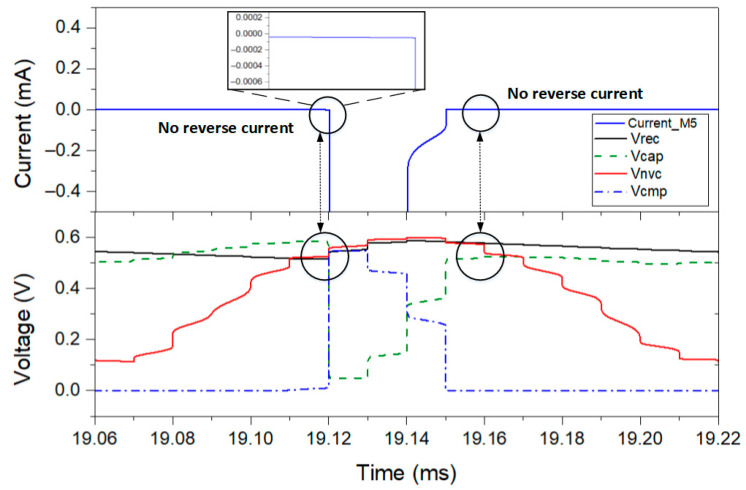
Simulated comparator behavior in steady state for $R_{\mathrm{LOAD}}=500\;Ω$ and $C_{\mathrm{LOAD}}=2\;µF$.

The reverse leakage current analysis is one of the most important analyses to make in CMOS rectifiers because it affects the power efficiency of the overall system. This reverse leakage current is dependent on the delay of the comparator and, consequently, of the discharging path of the active diode provided by the AVC. Therefore, the analysis of the transient performance of the comparator is shown in Figure 6. It presents the output voltage of the comparator ($V_{CMP}$), the input and output voltage of the active diode used to perform the comparison, the gate voltage of M5 ($V_{CAP}$), and the current that flows through the active diode ($I_{M5}$). As can be observed, the comparator immediately turns on the gate of the AVC transistor to create the discharge path when $V_{NVC}$ exceeds $V_{rec}$. At this stage, the current is flowing through M5, and $V_{CAP}$ is low, which leads to a low voltage drop because $V_{SG}$ is high. When $V_{NVC}$ drops below $V_{rec}$, the comparator then quickly turns off the AVC, and consequently the active diode. Thus, the proposed structure does not exhibit reverse leakage current that would degrade the PCE of the proposed rectifier.

### 4.3. Power Efficiency

**Figure 7:**
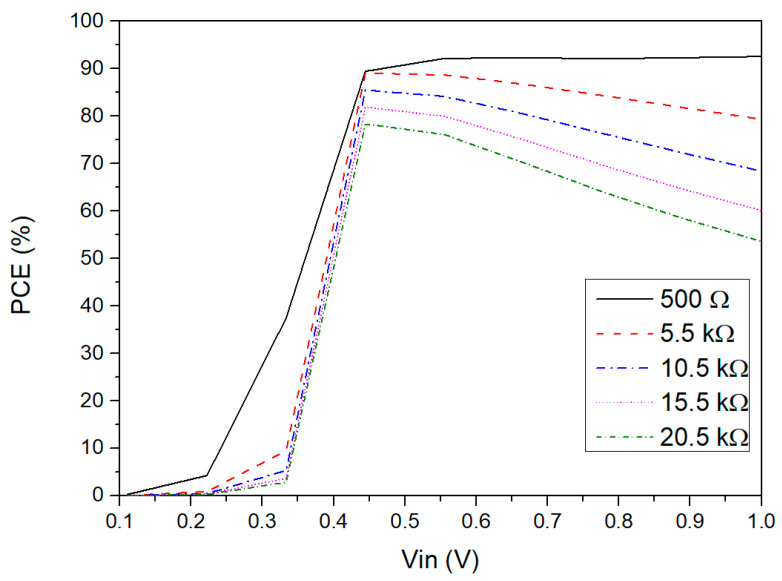
PCE versus input voltage amplitude simulated for different values of $R_{\mathrm{LOAD}}$

The simulated power efficiency versus input voltage amplitude for different load resistors is presented in Figure 7. The definition of PCE is shown in (5):

(5) $$PCE\;=\;\frac{\int_{t}^{t\;+\;T}V_{OUT}\left(t\right)\cdot I_{OUT}\left(t\right)dt}{\int_{t}^{t\;+\;T}V_{IN}\left(t\right)\cdot I_{IN}\left(t\right)dt}\cdot 100\%.$$

**Figure 8:**
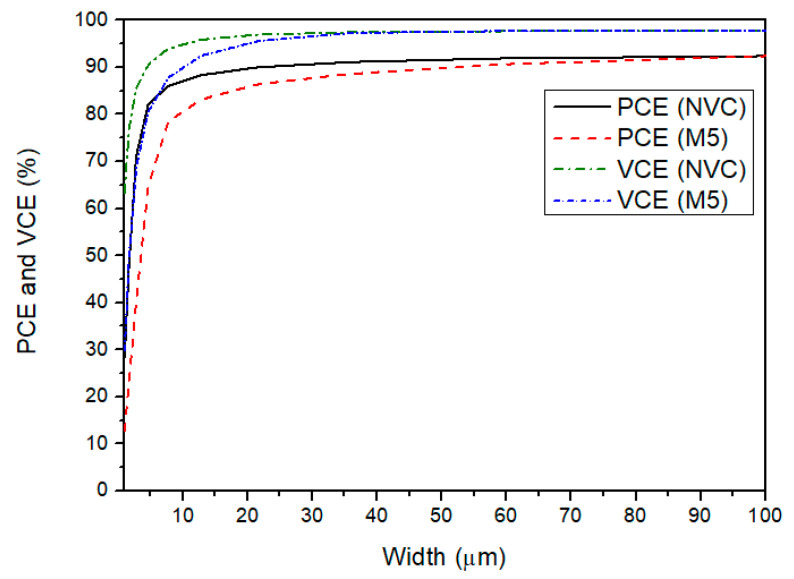
VCE and PCE features with the variation of the width of the NVC transistors and M5 (L = 0.13 µm).

The maximum PCE value of 94% can be found at 0.6 V for a $R_{\mathrm{LOAD}}\;$of 500 Ω. When $V_{in}$ is lower than this range, the PCE sharply decreases due to the low voltage efficiency, as noted in Section 4.1. Thus, the efficiency of the rectifier is poor in the ultra-low voltage range. Additionally, the PCE tends to decrease for higher input voltages because the power losses are mainly concentrated in the comparator. However, this case is not significant for ohmic loads lower than 15.5 kΩ. Moreover, for higher load resistors, the PCE tends to decrease due to the reduction of the output current, whereas the bias current that comes from the voltage source keeps almost constant. Regardless, from 0.45 V to 1 V, the power efficiency for low ohmic loads is considered as being good for this application. Additionally, the influence of the width of the NVC stage (M1–M4) and of M5 in both PCE and VCE can be observed in Figure 8. For this simulation test, the width of each stage was individually varied while the other was kept constant. This figure shows that the VCE and PCE features of both stages are at their maximum point for a width of 100 µm because the on-resistance of this transistor is directly influenced by the W/L ratio of the MOSFET. Even if the gate capacitance of M5 increases with the size, Figure 6 shows that the threshold cancellation circuit can drive this large transistor.

**Figure 9:**
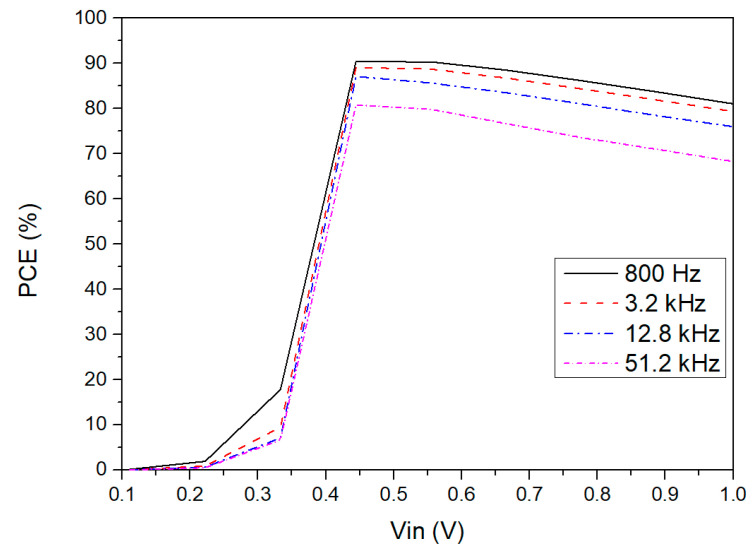
PCE versus input voltage amplitude simulated for different input frequencies for $R_{\mathrm{LOAD}}=5.5\;kΩ$

Figure 9 shows the power efficiency versus input voltage amplitude for different input frequencies. The load capacitor value was adapted to keep the output ripple voltage small depending on the input frequency. It is possible to observe that the proposed rectifier can achieve a high-power efficiency for low input frequencies in the operating voltage range. However, when the input voltage and frequency are high, the power efficiency tends to slightly decrease due to the power losses in the NVC and in the active diode, which in this case it is caused by the output signal of the comparator being too fast. Consequently, the working time of transistor M5 will be too short, which reduces the amount of power converted to the load. Nonetheless, at typical energy harvesting frequencies, the performance of the CMOS rectifier for the presented frequency range is suitable for this application.

**Figure 10:**
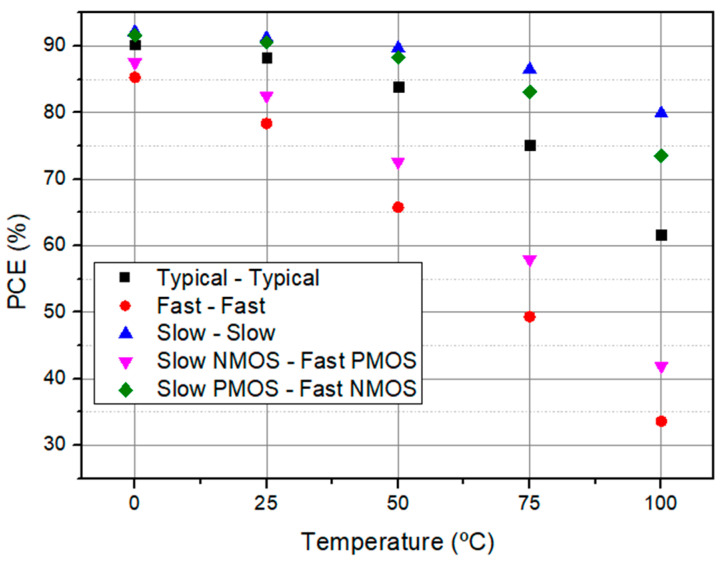
PCE variation with temperature depending on the process corner variation for a $R_{\mathrm{LOAD}}=5.5\;kΩ$

To prove the robustness of the proposed CMOS rectifier, it was tested through the four known process corners, such as the typical ones, fast, slow, slow NMOS, and fast PMOS, and fast NMOS and slow PMOS. Figure 10 presents the PCE plots with the variation of the temperature depending on the process corner. According to the simulation results, it can be observed that PCE tends to decrease when a fast PMOS is used due to the high speed of the active diode, which reduces the ON time of the rectifier. Consequently, the power transferred to the load is affected. Nevertheless, as long as the temperature rises, the power consumption of the rectifier also increases because the MOS threshold voltage is an exponential function of the temperature.

The performance comparison between this work and previous rectifiers is presented in Table 2. It shows that the proposed configuration can achieve higher VCE and PCE for a low voltage range. Even if the PCE in [29] is higher for a high ohmic load, for this application, it is only expected a low impedance of the electronics to be powered. Thus, the achieved VCE and PCE in this work are higher than those in the reported literature, highlighting its added value [15,28,30]. In addition, this work presents a wider input voltage range compared to the previously noted article. Therefore, it can be concluded that this rectifier can overcome the drawbacks of the structures discussed in Section 2, which means that this rectifier is very suitable for energy harvesting applications.

## 5. Conclusions

A highly efficient active CMOS rectifier suitable to be applied to vibrational energy harvesters was presented in this work. The proposed structure was designed in 130 nm CMOS technology, and the results showed a VCE of 99% and a PCE of 80–90% for a low operation voltage from 0.45 V to 1 V and for an operating frequency of 3.2 kHz, which proves the value of this work for a practical energy harvesting application. These features were achieved by combining an NVC with an active diode biased by a threshold cancellation circuit, which dynamically reduces the threshold voltage effect. Moreover, this structure avoids the reverse leakage current due to the use of a no-delay comparator, which was vital to reduce the power losses.

The research work focused on developing a highly efficient rectifier to be integrated into a PMC. It is believed that this structure will efficiently contribute to solving the battery limitation problems of the WSNs for an environmental monitoring application. Further work should focus on integrating the proposed structure in the PMC and respective testing in real environmental conditions.

## Figures and Tables

**Table 1:** Circuit transistor sizes.

	Unit Size (µm/µm)	Multiply Factor
M1/2/3/4	100/0.13	100
M5	100/0.13	50
M6/7/10/11/13/14/16	0.28/0.13	1
M7/9	20/0.13	1
M11/12	0.34/0.13	1

**Table 2:** Research comparison.

Ref.	Tech. (nm)	$f_{in}\;(\mathrm{kHz})$	$V_{in}\;(V)$	$R_{LOAD}\;(kΩ)$	$C_{LOAD}\;(µF)$	VCE (%)	PCE (%)
[15]	350	0.1	0.5	50	10	99	84
[28]	180	20	0.8	2	2	75	85
[29]	180	0.2	3	200	1	-	91.5
[30]	65	0.12	1.23	12	10	98	84
Proposed	130	3.2	0.45–1	5.5	2	99	80–90

## Data Availability

Not applicable.
